# *In silico* Effects of Synaptic Connections in the Visual Thalamocortical Pathway

**DOI:** 10.3389/fmedt.2022.856412

**Published:** 2022-04-05

**Authors:** Swapna Sasi, Basabdatta Sen Bhattacharya

**Affiliations:** Computer Science and Information Systems, Birla Institute of Technology and Science (BITS) Pilani, Sancoale, India

**Keywords:** thalamocortical model, *in silico* model, visual pathway, neural mass model, brain rhythm, frequency band transition, schizophrenia

## Abstract

We have studied brain connectivity using a biologically inspired *in silico* model of the visual pathway consisting of the lateral geniculate nucleus (LGN) of the thalamus, and layers 4 and 6 of the primary visual cortex. The connectivity parameters in the model are informed by the existing anatomical parameters from mammals and rodents. In the base state, the LGN and layer 6 populations in the model oscillate with dominant alpha frequency, while the layer 4 oscillates in the theta band. By changing intra-cortical hyperparameters, specifically inhibition from layer 6 to layer 4, we demonstrate a transition to alpha mode for all the populations. Furthermore, by increasing the feedforward connectivities in the thalamo-cortico-thalamic loop, we could transition into the beta band for all the populations. On looking closely, we observed that the origin of this beta band is in the layer 6 (infragranular layers); lesioning the thalamic feedback from layer 6 removed the beta from the LGN and the layer 4. This agrees with existing physiological studies where it is shown that beta rhythm is generated in the infragranular layers. Lastly, we present a case study to demonstrate a neurological condition in the model. By changing connectivities in the network, we could simulate the condition of significant (*P* < 0.001) decrease in beta band power and a simultaneous increase in the theta band power, similar to that observed in Schizophrenia patients. Overall, we have shown that the connectivity changes in a simple visual thalamocortical *in silico* model can simulate state changes in the brain corresponding to both health and disease conditions.

## 1. Introduction

Brain signals such as Electroencephalogram (EEG), Steady State Visually Evoked Potentials (SSVEP) and Local Field Potentials (LFP) are commonly used in research to understand and identify their correlation with the functional and behavioral states of the brain. Frequency domain analysis is an established, and by far the most popular way of analyzing these brain signals. Broadly, brain signal frequencies are clustered into five bands *viz*. Delta [δ: 0.5–4 Hz], Theta [θ: 4–8 Hz], Alpha [α: 8–12 Hz], Beta [β: 12–30 Hz] and Gamma [γ: >30 Hz] (1); in many studies, each band is further subdivided into sub-bands (2). Psychophysical studies associate these frequency band oscillations, often termed as “rhythms,” with different brain states in healthy adults. The δ frequency band is usually linked to deep sleep (3); θ oscillations in the neocortex are reported in memory and learning processes as well as in synaptic plasticity (4); α oscillations are most prominent during awake and resting state with eyes closed (1); β oscillations are associated with a high arousal state of the brain for example during sensorimotor activity (5); γ oscillations are associated with attention, consciousness, memory and perception (3). At the same time, alterations in brain rhythms are reliable neuromarkers in several neurological conditions. For example, a reduction in the β band power is a consistent observation in Schizophrenia patients (6); α rhythm slowing is a definitive marker in Alzheimer's (7) and chronic pain patients (8). The differences in the neuronal microcircuits influenced by their underlying synaptic connectivities and attributes are thought to underpin brain rhythmic changes (3); for example decrease in the white matter volume as well as in the overall GABA-ergic inhibition is reported from post-mortem studies of Schizophrenia patients (9). Although much is now known about the correlations between various brain oscillatory states and inter-region connections in the brain, it is hard to understand and identify exact pathway aberrations in disease condition, thereby making it difficult to provide effective treatment. In this regard, *in silico* models are used by many research labs to study and suggest effective treatment for brain disorders (10, 11). We have been using *in silico* models as a means to understand better the neural dynamics that underpin various brain oscillatory states. Here, we have used an enhanced version of an existing *in silico* model to understand the effects of synaptic connectivity changes on the oscillatory dynamics within the θ, α, and β frequency bands.

Recently, we have demonstrated the phenomenon of phase entrainment with periodic flicker visual input in an *in silico* model (12). The model consisted of three neural structures that lie in the brain visual pathway, *viz*. the lateral geniculate nucleus (LGN) of the thalamus that receive direct input from the retinal spiking neurons; Layer 4 (L4) of the primary visual cortex that are direct recipient of the visual information from the LGN; Layer 6 (L6) of the primary visual cortex that receive feed-forward input projections from the L4, and provide feedback to the LGN. This layout of the model is an abstraction of the thalamocortical loop in the brain that is essential for generating brain signals for example sleep rhythms (13). The intra- and inter-layer synaptic connectivity parameters for L4 and L6 in the model were based on the anatomical data from the cat primary visual cortex (14). However, the L6 also receives feed-forward projections from the LGN (15), which was not included in the model. In this work, we have presented an enhanced version of the previous model. First, we have included the feed-forward pathway from the LGN to the L6. Second, we have considered all synapses formed between any two populations in L4 and L6 irrespective of the layer where they were formed. For example, the excitatory projection neurons that have their perikarya in L4 also have dendritic projections in layer 5 (L5); similarly, the excitatory projection neurons that have their perikarya in L6 also project their axons to L5 and make pre-synaptic contact with L4 dendrites. This is unlike in our above-mentioned previous work (12), where we considered only those synapses which were made within the L4 and L6. Our modified approach is more biologically realistic and has resulted in an alteration of our base synaptic parameter values compared to that in the previous work. Third, we have addressed missing values in the anatomical data, the details of which are mentioned in section 2.

Our objective in this work is to study the synaptic connectivity changes that underlie alterations of the model signal power in the frequency spectrum for healthy, as well as neurological conditions. We have demonstrated the effect of “lesioning” (disconnecting) synaptic connections in specific pathways of the *in silico* model that shift the dominant oscillatory frequencies of the neuron populations. We have identified specific pathways in the intact visual thalamocortical loop that cause transitions from the base oscillatory frequency of the neural populations to a state with maximum power within the θ, α, and β bands. The identified synaptic pathways that underlie these state transitions agree with existing anatomical and physiological studies; we have discussed the details in section 4. To test the *in silico* model for simulating neurological conditions, we have demonstrated the decrease in β band power reported in Schizophrenia patients compared to controls (6, 9, 16). The critical factors to simulate the condition of Schizophrenia *in silico* are: an overall decrease in the excitatory and inhibitory membrane conductance; reduction in the feedforward excitatory projection signals from the LGN to L4. In summary, our results demonstrate the usability of a simple *in silico* model of the brain thalamocortical loop, informed by anatomical and physiological synaptic layout and connectivities, to understand the neural pathways that underpin brain oscillatory changes, as well as to validate and inform psychophysical studies.

The layout of this article is as follows: In section 2, we detail the biological basis of the model and the anatomical sources that inform the parameterization of the model synaptic pathways. The simulation methods are also specified. The simulation results and observations are mentioned in section 3. In section 4, we make a detailed discussion of our observations in context to findings from anatomical, physiological and psychophysical research. Concluding remarks are mentioned in section 5.

## 2. Materials and Methods

**Figure 1:**
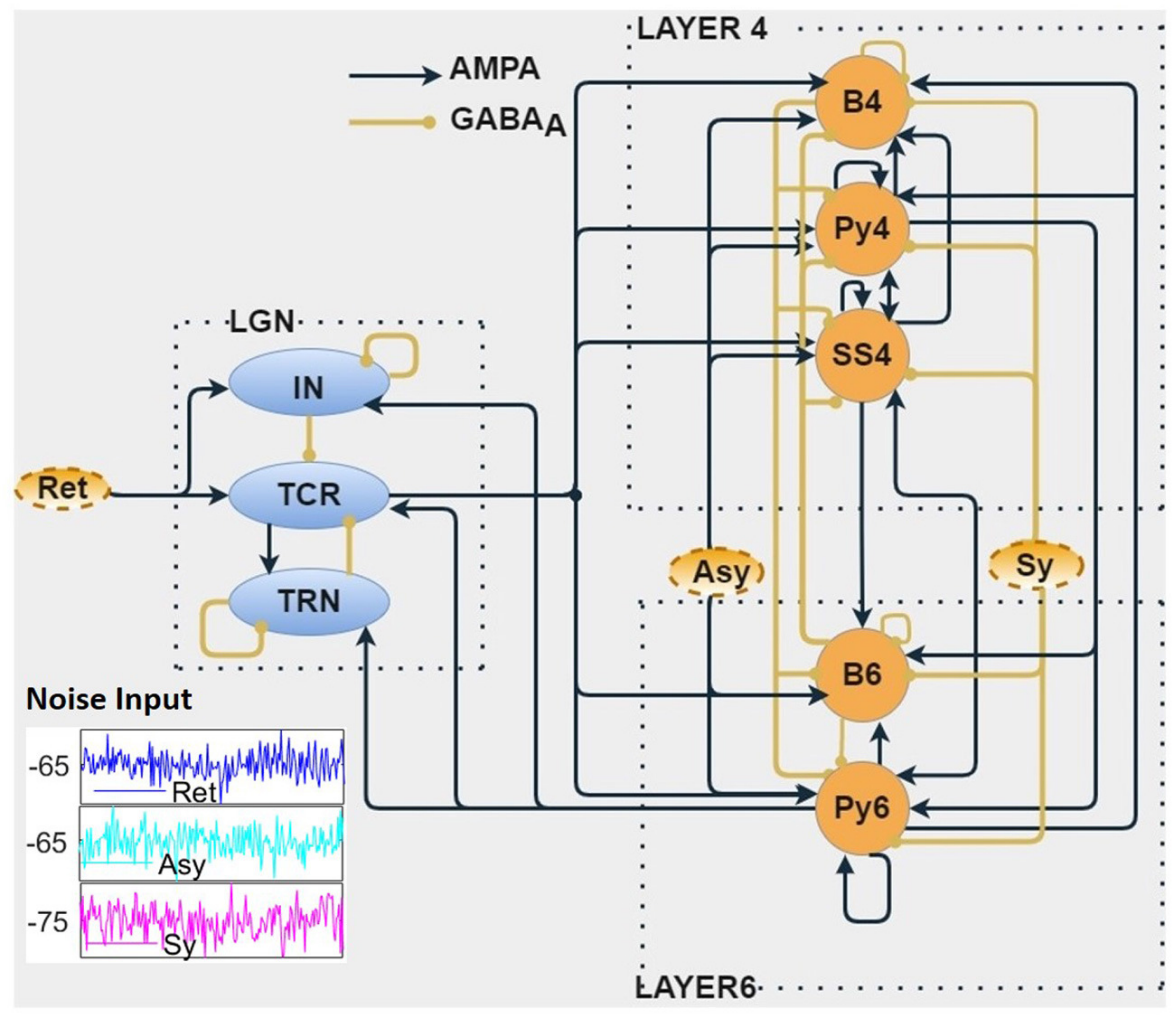
The biologically inspired population neural network consists of three modules *viz*. Layer 4 (L4), Layer 6 (L6) of the primary visual cortex, and the visual thalamus [Lateral Geniculate Nucleus (LGN)]. The LGN neural populations receive inputs from the retina (Ret). The Relay cells (TCR) of LGN project on to the L4 and L6 populations. Connection between L4 and L6 are bidirectional. The L6 populations project back to the LGN, thus forming a closed loop. Both cortical layers are provided with noisy inputs simulating excitatory (Asy) and inhibitory (Sy) projections from other parts of the cortex. The outputs of this *in silico* model are the time series responses of excitatory populations from each of the three modules *viz*. TCR cells of the LGN, Py4 cells of L4, and Py6 cells of L6. The excitatory and inhibitory synaptic connections are shown as AMPA-based and GABA_*A*_-based, respectively. (inset) Show-casing samples of random noise inputs for 200 ms.

The thalamocortical model is shown in Figure 1. In section 2.1, we present the biological basis of the model synaptic layout and the data sources for the synaptic connectivity parameters used in the model. In sections 2.3 and 2.4, we present the parameterization and simulation methods, respectively, of the model.

### 2.1. Background

The mammalian neocortex is known to have six stratified layers with distinct cell types (17). The direction of the layers is from the inside of the skull (Layer 1) toward the deeper brain. In addition, the neocortex is also known to have a columnar structure, where each adjacent column consists of the six cell layers. The primary information carrying excitatory cell populations of the neocortex are the Pyramid cells. In contrast, there are a myriad of inhibitory cell types that are mainly identified by their physical shapes, and are broadly classified as Basket and non-Basket cells. The layered and columnar architecture, along with the different cell types are largely similar in the different parts of the neocortex even if they cater to different brain functions; the main difference is in the synaptic connectivities that specific parts of the neocortex forms with the subcortical structures as well as with other parts of the neocortex.

Our interest in this work is the primary visual cortex, that is also referred to as V1, or striate cortex in primates, and area 17 in cats. The visual signals from the eye are projected on to the LGN of the thalamus, which relays the information on to the V1 for further processing. For a long time, the main function of the LGN was thought to be to relay retinal information to V1. Subsequent research has shown that the LGN also receives influential feedback from the V1 (18), thus making the LGN a critical component for generation of brain rhythms. The main recipient of the LGN outputs are the L4 and L6 of V1; the feedback to the LGN is primarily from the L6 (15). This is the fundamental structure of our model layout as shown in Figure 1, consisting of three distinct modules *viz*. LGN of the thalamus, and L4 and L6 of the V1. The excitatory populations in L4 and L6 are the Pyramidal cells Py4 and Py6, respectively; the inhibitory cells in the respective layers are the basket cells B4 and B6 (for brevity, we did not consider the Non-Basket cells, see section 2.3 for an explanation). L4 also has an excitatory interneuron population *viz*. spiny stellate cells (SS4). The LGN is represented by the excitatory thalamocortical relay cells (TCR) and the inhibitory interneurons (IN). Due to the role of the inhibitory thalamic reticular nucleus (TRN) as a “gateway” in the connectivity between thalamus and cortex, it is generally modeled as a part of the thalamus. The mathematical equations that define the model structure and dynamics are discussed in section 2.2.

The outputs of the model that are studied for oscillatory behavior as observed in EEG and LFP are those from Py4, Py6, and TCR. However, readers may note that we are in no way implying a direct equivalence of our model output with EEG or LFP recordings. Rather, our assumptions are based on studies that show that the current source density of LFP recordings from V1 following visual stimuli have origins in L4 (19), which are further driven by thalamocortical afferents. Thus, our model outputs are representative of physiological recordings from cortical layers that underlie the occipital lobe, responding to visual inputs that are relayed by the LGN.

### 2.2. Model Equations

The mathematical equations that define our *in silico* model structure and dynamics are given below:


(1) $$\begin{array}{lll} {\left[T\right]}_{u} & = & \frac{T_{max}}{1+e^{-\frac{\left(V_{u}\left(t\right)-\theta_{u}\right)}{\sigma }}} \end{array}$$

(2) $$\begin{array}{lll} \frac{dr_{uv}^{\text{Ψ}}\left(t\right)}{dt} & = & \alpha^{\text{Ψ}}{\left[T\right]}_{u}\left(1-r_{uv}^{\text{Ψ}}\left(t\right)\right)-\beta^{\text{Ψ}}r_{uv}^{\text{Ψ}}\left(t\right) \end{array}$$

(3) $$\begin{array}{lll} I_{uv}^{\text{Ψ}}\left(t\right) & = & C_{uv}^{\text{Ψ}}g_{max}^{\text{Ψ}}r_{uv}^{\text{Ψ}}\left(t\right)\left(V_{v}\left(t\right)-E_{rev}^{\text{Ψ}}\right) \end{array}$$

(4) $$\begin{array}{lll} \kappa \frac{dV_{v}\left(t\right)}{dt} & = & -\underset{uv}{\sum }\left(I_{uv}^{\text{Ψ}}\left(t\right)\right)+I_{v}^{lk}\left(t\right) \end{array}$$

(5) $$\begin{array}{lll} I_{v}^{lk}\left(t\right) & = & g_{v}^{lk}\left(V_{v}\left(t\right)-E_{v}^{lk}\right) \end{array}$$

where *u* ∈ {TCR, TRN, IN, Py4, SS4, Py6, B4, B6, Asy, Sy, Ret}, *v* ∈ {TCR, TRN, IN, Py4, SS4, Py6, B4, B6} are the pre-synaptic and post-synaptic neuronal populations, respectively; Ψ ∈ {AMPA, GABA_*A*_} represents the neurotransmitter-receptors on *v* and can be of excitatory (*AMPA*) or inhibitory (*GABA*_*A*_) nature; [*T*]_*u*_ is the neurotransmitter concentration in the synaptic cleft and is expressed as a sigmoid function dependant on the pre-synaptic membrane potential *V*_*u*_ and the maximum neuronal concentration *T*_*max*_ ≈ 1*mM* (20); θ_*u*_ represents the threshold voltage where [*T*]_*u*_ = 0.5 *T*_*max*_ and σ is the slope of the sigmoid; $r_{uv}^{\text{Ψ}}$ is the proportion of open ion-channels due to the pre-synaptic neurotransmitters binding on the post-synaptic neurotransmitter-receptors on the ensemble membrane of *v*; α^Ψ^ and β^Ψ^ are forward and reverse rates of chemical reaction, respectively; $g_{max}^{\text{Ψ}}$ is the maximum conductance of the ensemble post-synaptic membrane due to opening of ion channels, resulting in the post-synaptic current $I_{uv}^{\text{Ψ}}\left(t\right)$; $C_{uv}^{\text{Ψ}}$ is the connectivity strength of any synapse from *u* to *v*; *V*_*v*_(*t*) is the ensemble post-synaptic membrane voltage, $E_{rev}^{\text{Ψ}}$ is the reverse potential corresponding to the neurotransmitter-receptor Ψ; κ is the membrane capacitance (pF); $I_{v}^{lk}$ is the ensemble leak current; $g_{v}^{lk}$ and $E_{v}^{lk}$ are the maximum leak conductance and leak reversal potential, respectively, of *v*. All the model parameters used in above equations are given in Table 1.

**Table 1:** The parameters used in (1) – (5) that define the model framework.

**(I) Neurotransmission parameters**									
**Parameters**	**Value**	**Synapse type (Ψ)**							
α^Ψ^((*mM*)^−1^.(*s*)^−1^)	100	AMPA,GABA_*A*_							
β^Ψ^(*s*^−1^)	50	AMPA							
	50	AMPA							
	40	GABA_*A*_							
$g_{max}^{\text{Ψ}}\left(\mu S/cm^{2}\right)$	1000	GABA_*A*_							
	300	AMPA(Ret to TCR)							
	100	AMPA(Ret to IN) (TCR to TRN)							
$E_{rev}^{\text{Ψ}}\left(mV\right)$	0	AMPA							
	–85	GABA_*A*_(inter-population)							
	–75	GABA_*A*_(recurrent)							
**(II) Cell Membrane parameters**									
**Parameters**	**Ret**	**TCR**	**IN**	**TRN**	**Py4, Py6**	**B4, B6**	**SS4**	**Asy**	**Sy**
*g*^*lk*^(μ*S*/*cm*^2^)	X	10	10	10	10	10	10	X	X
*E*^*lk*^(*mV*)	X	−55	−72.5	−72.5	−55	−72.5	−55	X	X
*V*_*rest*_(*mV*)	−65	−65	−75	−85	−65	−85	−65	−65	−75

‘*X' indicates that these values are not used or required in the model*.

### 2.3. Methods of Parameterizing Synaptic Connectivity

For parameterizing the V1 modules in our model, we follow the comprehensive anatomical study of the cat's primary visual cortex by Binzegger et al. (14). They provide the connectivity map by reconstructing complete axons, boutons and dendrites of 39 single neurons from intra-cellular *in vivo* recordings of the cells. Our interest is in the L4 and L6 and their afferent connections. Toward this, we have extracted all the relevant data from Tables 1–5 in Binzegger et al. (14) into Table 2, which shows the absolute number of synapses formed by each presynaptic and postsynaptic cell types in each of the cortical layers. It is based on the modified version of Peter's rule (21) where (14) assume that synaptic densities on dendrites are independent of the cell types. In addition, ours is a population model that assume a mesoscopic neural population as a point neuron, and the afferent synaptic connectivity parameters of a post-synaptic population are expressed as percentages. For example from Table 2, we calculate the synaptic connectivities as follows:

- Let *X* and *Y* be any pre- and post-synaptic population, respectively, in Table 2.
- Let *N* be the sum of the synapses that all pre-synaptic populations of *Y* form with the cell body and dendrites of *Y* in all the layers. This can be obtained by adding all elements of the row vector corresponding to *Y* in Table 2.
- Let *M* be the sum of all the synapses that *X* make on to the cell body and dendrites of *Y* in all the layers. This is the value at the intersection of a row vector *Y* and a column vector *X* in Table 2.
- Then, the synaptic connectivity parameter C from *X* to *Y* is expressed as follows:

(6) $$\begin{array}{l} C_{X\to Y}=\frac{M}{N} \end{array}$$

**Table 2:** The table shows the absolute number of synapses formed by each presynaptic and postsynaptic cell types in each of the cortical layers.

**Pre → Post↓**	**Py2/3**	**SS4(L4)**	**SS4(L2/3)**	**Py4**	**Py5(L2/3)**	**Py5(L5/6)**	**Py6(L4)**	**Py6(L5/6)**	**X/Y**	**Asy**	**B2/3**	**B4(B6)**	**B5**	**Sy**	**db2/3**
B4(B6)	188.3	355.2	121.7	135.8	36.4	1.6	980.2	0.8	52.2	793.1	16.8	349.1	0	173.1	26.1
Py4	815.2	583.6	243.3	282.5	127.7	4.5	1602.4	5.3	84.9	1957.3	57	472.8	2.6	388.4	75.7
Py6(L4)	709.8	294.4	131.1	213.3	154.1	170.8	711	452.4	54.7	2518.6	20.6	136.9	26.6	744.5	24.1
Py6(L5/6)	449.4	80.9	83	123.7	58.9	274.5	160.6	740	37.1	3406.8	22.5	23.4	21.4	917.5	21.3
SS4(L2/3)	277.6	562.2	190.4	212.3	55	2.5	1551.2	1.3	82.6	1255.8	22.1	462	0	273.8	40
SS4(L4)	157.4	687.8	212.5	236.7	44.8	3.1	1895.3	1.6	101.2	1540.1	10.9	529.7	0	333.4	37.1

*It is based on the modified version of Peter's rule where (14) assume that synaptic densities on dendrites are independent of the cell types. Inter- and intra-layer afferents to the L4 and L6 populations are extracted from (14) [see Tables 1-5 in (14)]. Py, B, SS and db indicate the Pyramid, Basket, Spiny Stellate and double bouquet (non basket variety) neuron populations, respectively, where the numeric suffix represents the cortical layer index; Asy and Sy are the Asymmetric (excitatory) and Symmetric (inhibitory) afferents, respectively, from other regions of the cortex; X/Y represent the afferents from the LGN. Readers may note that non-basket cells are not included in this work as the available data do not show any efferent connections of this variety in L4 and L6; thus, our results remain unaffected by this abstraction*.

The synaptic connectivity parameters thus calculated are shown in Table 3 and are considered as the base state parameters of the model. However, there are gaps in the collected data due to obvious constraints in neurophysiological and anatomical studies; we note that the B6 connections are not documented in Binzegger et al. (14). We have filled these missing values by assuming the same values as corresponding pathways in B4, and then converting to percentage form by using layer specific values in Equation (6). However, the Py6 mean membrane potential was in an “up state” at -20 mV compared to ≈ −60 mV range for other populations. To induce a relative hyperpolarisation in Py6, we increased the B6 to Py6 synaptic connectivity by ten times of its initially set value. This reduced the mean membrane potential of Py6 to −60 mV, and was within the subthreshold oscillation regime as demonstrated in other populations. The intra-LGN connections are as in our previous work (2, 12); the TCR and IN afferent data are obtained from the cat dorsal LGN (22); the TRN afferent data are based on the rat dorsal LGN (23). The feedback from L6 to LGN are derived from Van Horn et al. (22), Jones (23) and are as in Sen Bhattacharya et al. (2). The feed-forward connectivity of LGN to L4 and L6 populations are derived from Binzegger et al. (14). While there are non-basket afferents that project to the L4 and L6 populations as indicated in Table 2, we could not find any efferent connections of these populations in our data source (14). Thus, modeling the non-Basket cells for L4 and L6 would require filling in the missing data, which would increase the number of free parameters in the model. We have ignored the non-Basket cells in this work, and leave this as a future prospect.

**Table 3:** The synaptic connectivity parameters derived from Binzegger et al. (14) and set as the base state of the model.

**Pre → Post↓**	**TCR**	**TRN**	**IN**	**Py4**	**SS4**	**Py6**	**B4**	**B6**	**Asy**	**Sy**	**Ret**
TCR	-	23.175*	7.725*	-	-	62*	-	-	-	-	7.1*
TRN	35*	20*	-	-	-	50*	-	-	-	-	-
IN	-	-	23.6*	-	-	29*	-	-	-	-	47.4*
Py4	1.2	-	-	4	11.6	22.5	6.6	6.6	27.3	5.5	-
SS4	1.6	-	-	3.9	14.1	29.4	8.5	8.5	23.8	5.2	-
B4	1.5	-	-	3.8	13.4	27.5	9.8	9.8	22.2	4.9	-
B6	1.5	-	-	3.8	13.4	27.5	9.8	9.8	22.2	4.9	-
Py6	0.8	-	-	4.3	4.6	16	1.3	**13**	45.8	12.9	-

The parameters set to ‘*' are as in our previous models (2, 12). All other parameters are derived from Table 2, and are a part of model enhancement; the parameter marked in bold is a model hyperparameter and set by trial and error to set the membrane potential of Py6 to ≈-60 mV (see section 2.3 for details). The ‘-' indicate an absence of anatomical connection.

The model presented in this work builds on top of an existing model that was used as a tool to validate phase entrainment in SSVEP signals. We have introduced several model components that make this version more biologically plausible. First, we have introduced the TCR to L6 projections that form an integral part of the LGN projections on V1 in the brain (15). Second, all synapses between L4 and L6 that form outside of these two layers are used to compute the synaptic connectivity parameter values. This is unlike in the previous model where synapses formed only within L4 and L6 are considered. Third, the Asymmetric (Asy) and Symmetric (Sy) noise inputs to the L4 and L6 are derived from the data in Binzegger et al. (14), unlike in the previous model, where the Asy and Sy sources are combined for all populations.

### 2.4. Methods of Simulation

The *in silico* model is simulated on Matlab (™Mathworks Inc.). Total simulation duration is 120 s with a simulation timestep of 1 ms. The differential equations governing the model dynamics are solved in MATLAB using custom-written 4th/5th order Runge-Kutta-Fehlberg method (RKF45).

The noise inputs to the network populations, *viz*. retinal (Ret) input to the LGN, asymmetrical (Asy - excitatory) and symmetrical (Sy - inhibitory) inputs from other cortical areas to L4 and L6, are simulated using the *randn* inbuilt function in Matlab that generates a uniform distribution. This white noise is then normalized to a mean of -65 mV for Ret and Asy, and -75 mv for Sy, with standard deviation of 2 mV.

For consistency of results, the output voltage timeseries of each neuron population is averaged over 20 trials, where each trial is simulated with different white noise input. For frequency domain computation, the output timeseries is clipped to after and before 1 s of the start and end times, respectively, to avoid any transient effects. This clipped signal is then filtered using Butterworth bandpass filter with cut-off frequencies at 1 and 50 Hz. To calculate the power spectral density, Welch periodogram is used with a sampling frequency of 1,000 Hz and a frequency resolution of 0.25 Hz.

For calculating statistical significance of the difference observed in the output of control and Schizophrenia conditions of the model, *ttest* function of Matlab is used. *ttest*(*x, y*) returns the *P*-value using the paired-sample t-test at a 5% significance level. So if *P* < 0.05, then the result is considered statistically significant.

Frequency bands are computed with the following lower and upper bounds: theta (θ): 3.75–7.5 Hz, alpha-low (α-L): 7.75–11 Hz, alpha-high (α-H): 11.25–13.5 Hz, beta-low (β-L): 13.75–20.5 Hz, beta-high (β-H): 20.75–30.5 Hz. The bar plots are used for visualizing the results, where the power at all the frequency components that lie within the upper and lower bounds defined above for each frequency band are summed. Thus, each bar represents the area under the power curve for a specific frequency band.

The periodic inputs to the Ret populations to simulate SSVEP-like brain signals in the network are custom-written in Matlab to generate a pulse signal with on-time of 1 ms, generated at frequencies 1–30 Hz and amplitudes ranging from 5–10 mV, which is then superimposed on the above-mentioned white noise input.

Lesioning is a common technique in experimental literature to test the effect of one neural population on the other (24). In this study, we simulate lesioning by removing the synaptic connectivity between any two populations of interest.

## 3. Results

### 3.1. The Base State Behavior

**Figure 2:**
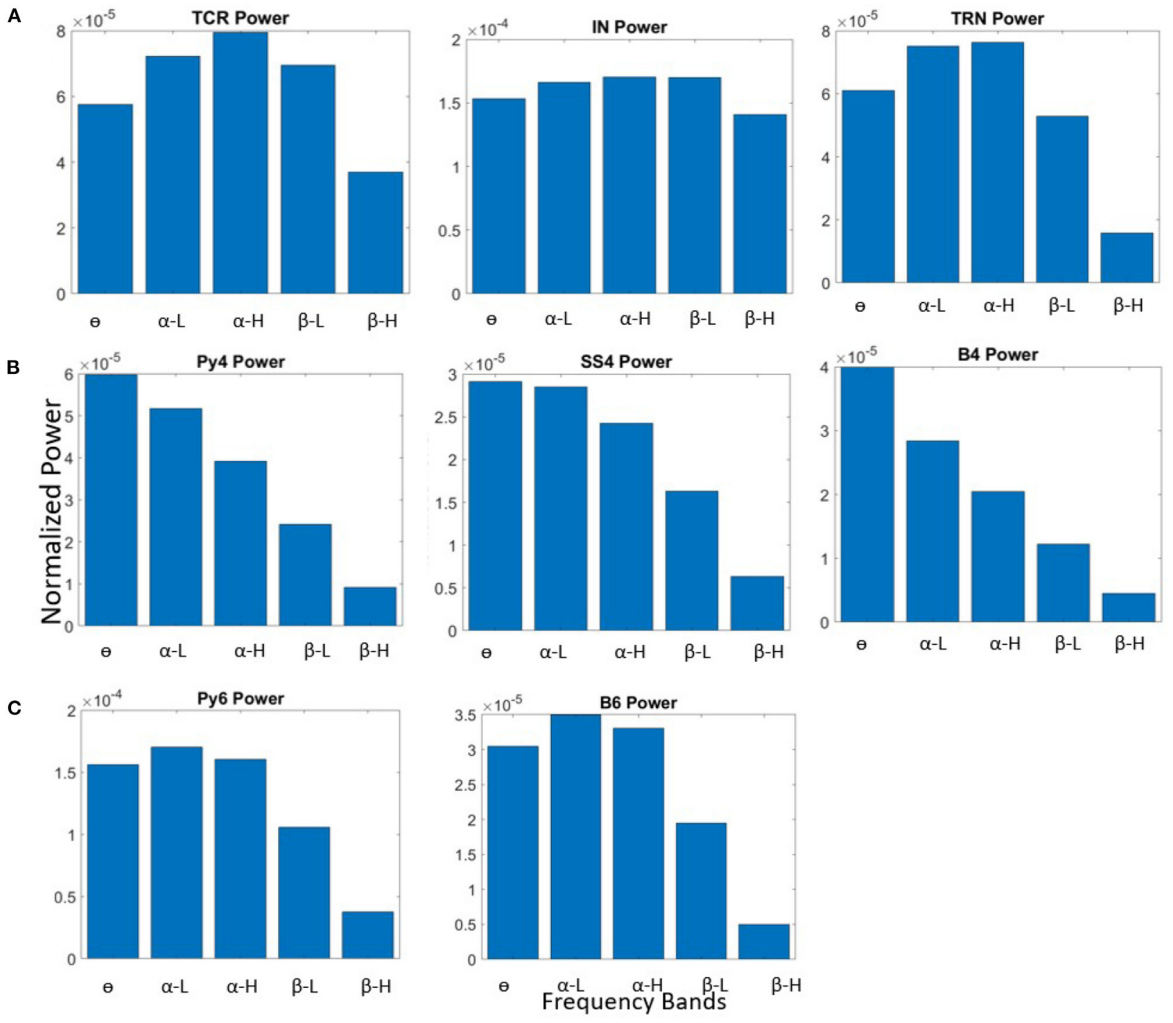
Bar plots showing power within frequency bands for all neural population in the **(A)** LGN, **(B)** L4, and **(C)** L6, when the synaptic connectivity parameters are set to their base values shown in Table 3.

The model behavior with the base state parameters in Table 3 is shown in Figure 2. All populations of the LGN and L6 have dominant frequency within the α band, and all the populations in L4 have θ band dominant frequency. The membrane potentials reflect the noise inputs to the model (not shown here). We consider this as the base (reference) state of the network, which correspond to “controls” in experimental studies consisting of normal healthy adults. The effects of changing specific synaptic connectivities in the network are presented with reference to this control state.

### 3.2. Lesioning the LGN

**Figure 3:**
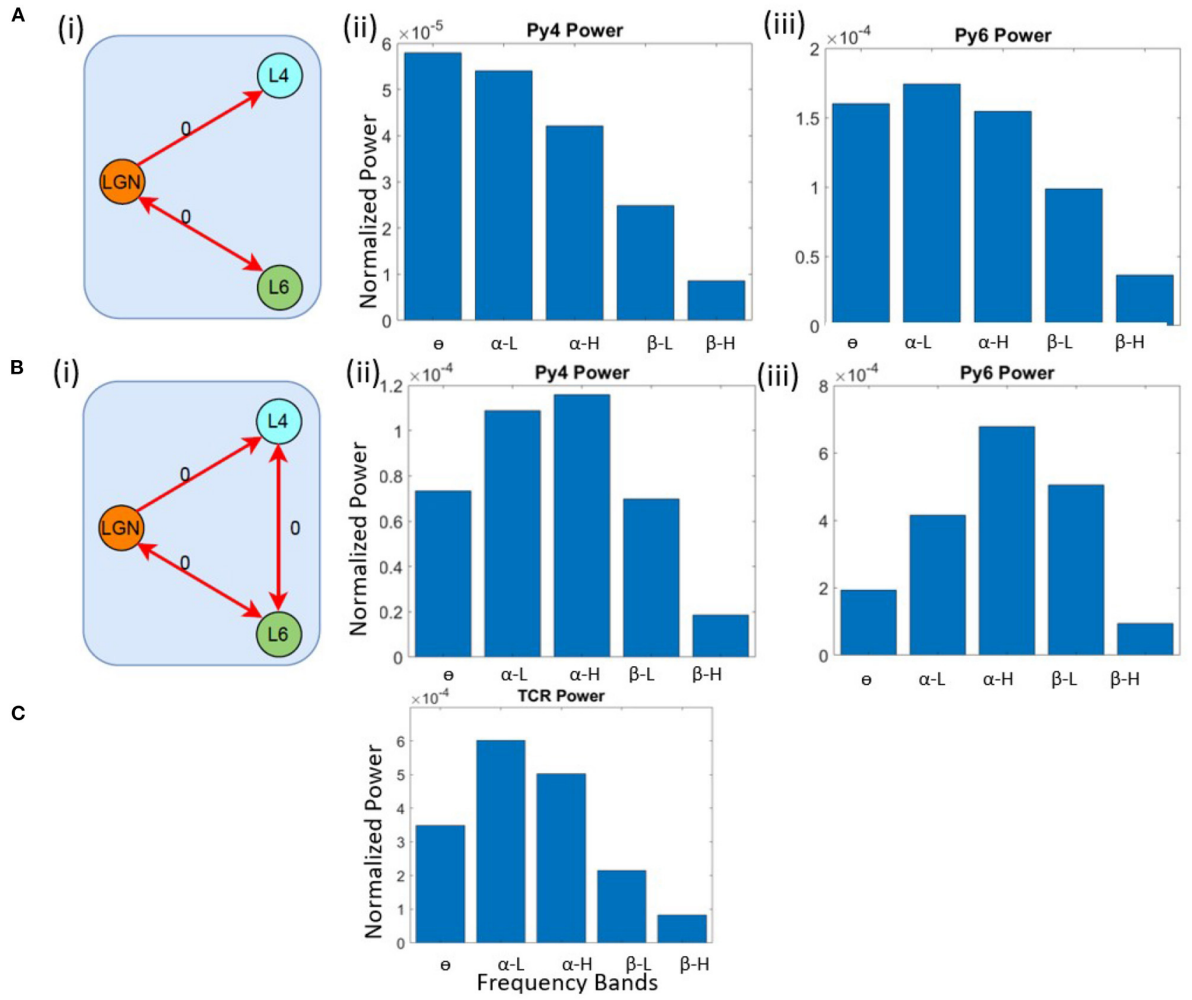
Schematic diagrams showing lesioning of network populations for identifying the **(A)** (i) α and **(B)** (i) θ band sources. The red links indicate lesioned synaptic pathways;the bidirectional links indicate closed loop connection between two populations; the number 0 beside each link highlight the connectivity parameter value to simulate a lesion in the synaptic pathway. Bar plots showing dominant power in the frequency spectrum for **(A,B)** (ii) Py4, **(A, B)** (iii) Py6, **(C)** TCR populations.

First, we checked the source of α peak in the TCR and Py6 outputs. We lesioned the LGN from the V1 layers by removing all feed-forward and feedback connections from and to the LGN, respectively. The inter-layer connections between L4 and L6 are kept intact, as are all intra-layer connections. The schematic of this network is shown in Figure 3A(i). The outputs of the excitatory projection neurons in the V1 layers *viz*. Py4 and Py6 are shown in Figures 3A(ii),(iii); the TCR output is shown in Figure 3C. When compared with their respective base state oscillations shown in Figure 2, we note that the dominant frequencies of the V1 populations continue to be the same, implying no dependency of L4 and L6 on LGN. However, the TCR output peak frequency move left on the frequency spectrum from α-H to α-L, indicating the effect of lesioning the feedback from Py6 population. Thus, when the intra-LGN connectivity parameters are set to their base values, the independent oscillation of the TCR has peak power in the α-L band, with a peak frequency observed at around 10 Hz.

Next, we wanted to identify the source of θ peak frequency in L4. The LGN remain lesioned as above. In addition, we have lesioned the inter-layer connectivity between L4 and L6. Figure 3B(i) shows the schematic diagram of this lesioned network, and Figures 3B(ii),(iii) show the frequency domain behavior in this state. We note that the maximum power of Py4 output is now within the α band, where the peak frequency is within the α-H band. The dominant frequency of oscillation in Py6 is now within the α-H band. We make several observations from these results: first, the Py4 independently oscillates within the α band and the slowing of its frequency response in the intact state of the network is caused by the afferent projections from the L6 populations; second, the inter-layer dynamics between L4 and L6 effects a “slowing” (left shift) of the frequency spectrum in the intact network state; third, and most importantly, both LGN and V1 layers can act as sources of α rhythm generation. These observations are discussed in context to physiological findings in section 4.

### 3.3. Simulating Frequency Band Transition

Frequency band transitions in the brain signals of a healthy adult are indicators of the different brain states. Here, we identify the synaptic connectivities that can simulate frequency band transitions in the intact network state, thus simulating control states, as in experimental studies, corresponding to specific oscillatory frequencies.

#### 3.3.1. Transitioning to the Theta Band

**Figure 4:**
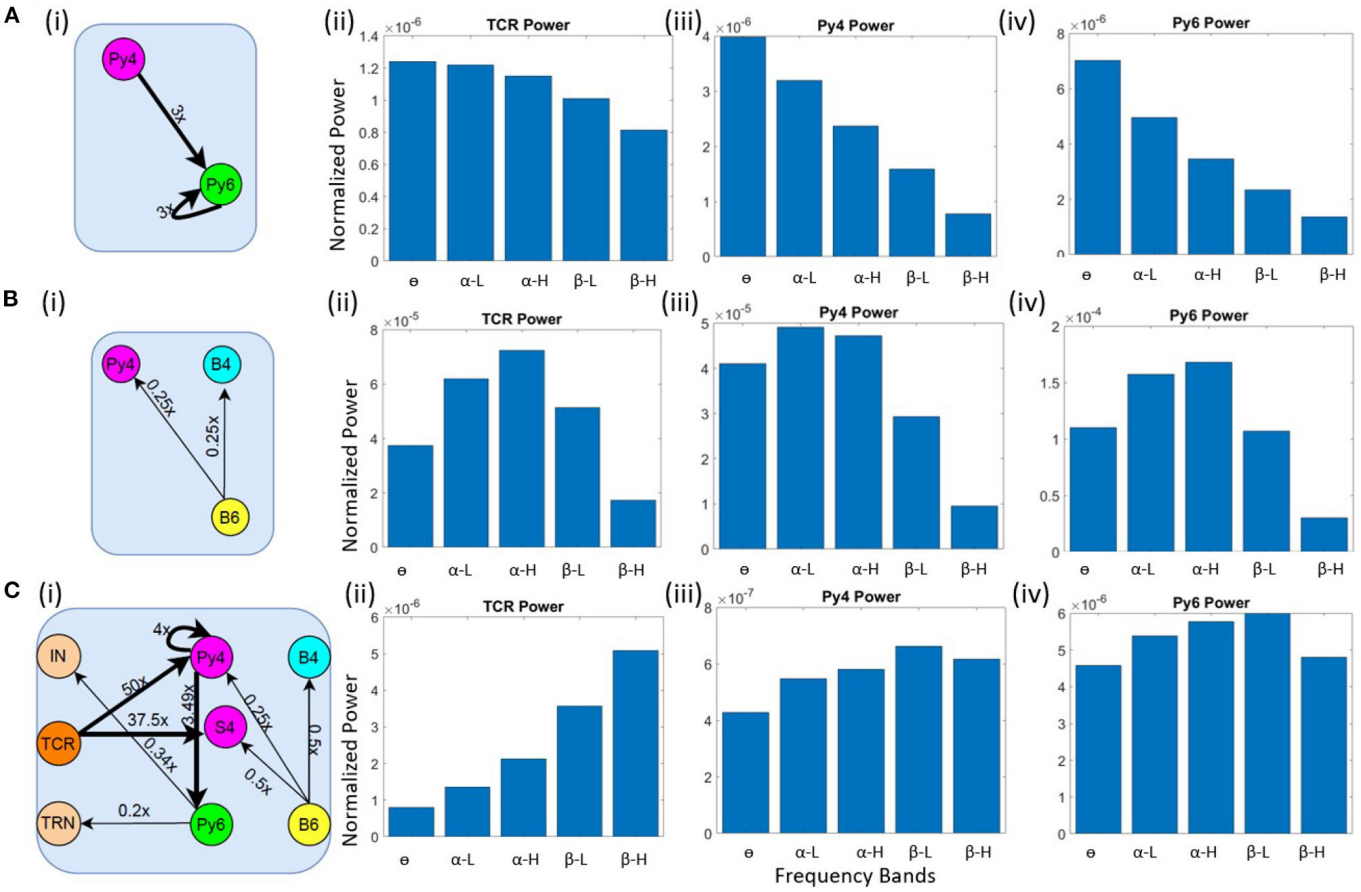
Schematic diagrams showing changes in synaptic connectivity parameters that effect a model state transition into the **(A)** (i) θ, **(B)** (i) α and **(C)** (i) β bands. The thick black links indicate increased synaptic connectivity, and *thin black links* indicate decreased synaptic connectivity from their base values; the percent decrease is mentioned beside each link, where *x* is the base connectivity parameter for that link. The dominant power within the **(A)** θ, **(B)** α and **(C)** β bands for (i) TCR, (ii) Py4 and (iii) Py6 populations shown as bar plots of the integrated power within each frequency band.

Our objective here is to understand the changes in synaptic connectivity that effected a dominant power within the θ band for the excitatory projection neurons. Our results show that this happens when there is an overall increase in the excitation of the Py6 population. Specifically, the feed-forward excitatory projection from Py4 to Py6, as well as the recurrent excitatory connection of the Py6 population, were increased by a factor of three. Note that Py4 is already in its base state dominant frequency of θ band. The schematic of the synaptic connectivity changes are shown in Figure 4A(i). The frequency response in Figures 4A(ii)–(iv) show the dominant frequency within the θ band for all excitatory projection neuron populations.

#### 3.3.2. Transitioning to the Alpha Band

As discussed in section 3.2, the θ band dominance in Py4 is shown to be caused by the L6 afferents; lesioning the afferents moved the Py4 dominant oscillations into the α band. Taking cue from this observation, we decreased the inhibitory projections from L6 to the Py4 and B4 populations of L4 by ≈ 25% as shown in the schematic in Figure 4B(i). The frequency domain response of the populations are seen to be α dominant in the Figures 4B(ii)–(iv). Overall a decrease in the inhibitory feedback from L6 to L4 effects a peak within the α band for Py4. The Py6 and TCR population dynamics were already dominant within the α frequency band in the network base state.

#### 3.3.3. Transitioning to the Beta Band

The connectivity scheme for transitioning into the β band from the respective base states of all neuron populations is shown in Figure 4C(i) and can be summarized as follows: (a) The feedforward excitatory connectivities TCR→(Py4,S4), and Py4→Py6 are increased; (b) the excitatory recurrent connection of Py4 is increased; (c) the inhibitory feedback from L6 to L4 is decreased; (d) the excitatory feedback from L6 to the inhibitory populations of LGN are decreased. The exact changes in the connectivity parameters is mentioned in Figure 4C(i). The frequency domain response in Figures 4C(ii)–(iv) show β dominance in the TCR, Py4 and Py6 populations. The changes (a)–(c) imply an overall increase in the excitation of the L4 populations; the change in (d) implies a reduced inhibition in the TCR population. Overall, we observe an increased excitation in the LGN and the L4 playing central role in the β band transition from the base state of the network.

Upon testing our network for frequency band transitions as seen in healthy brain signals, we wanted to test the efficacy of the model output as a neuromarker of frequency band transitions in Schizophrenia. Toward this, we aimed to understand specific connectivity pathways that generated known frequency band transitions observed in Schizophrenia patients. We present our findings in the following section 3.4.

### 3.4. A Case Study of the Frequency Band Alterations in Schizophrenia Patients

**Figure 5:**
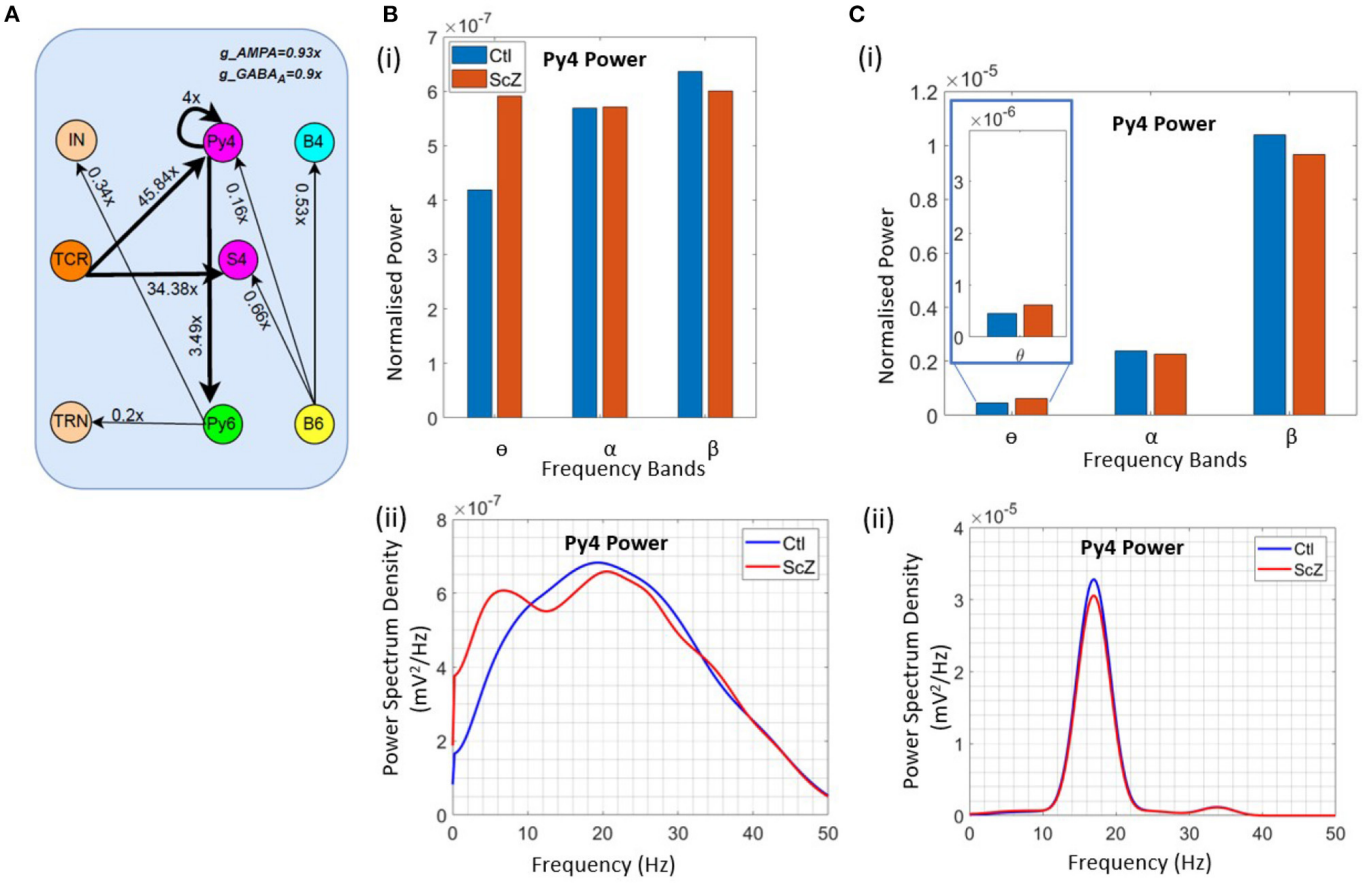
Results showcasing Py4 output changes on simulation of Schizophrenia (ScZ) condition in the model with respect to a control (Ctl) condition. **(A)** Schematic diagrams showing changes in synaptic connectivity parameters that simulate the condition of ScZ in the model when the Ctl state is in the beta peak oscillatory state. The thick black links indicate increased synaptic connectivity, and *thin black links* indicate decreased synaptic connectivity from their base values; the percent decrease is mentioned beside each link, where *x* is the base connectivity parameter for that link. **(B)** (i) Bar and (ii) line plots showing a significant (*P* < 0.001) increase in power within the θ band, and a significant (*P* < 0.001) decrease in power within the β band for the simulated ScZ condition compared to Ctl. **(C)** (i) Bar plots and (ii) line plots corresponding to SSVEP in simulated ScZ condition when the LGN is fed with a 17 Hz periodic flicker stimulus. The zoomed inset shows a significant (*P* < 0.001) increase in power within the theta band and a significant (*P* < 0.001) decrease in beta power. The line plot further confirms the decrease in power of the first harmonic for ScZ condition.

To simulate the condition of Schizophrenia, we set the β oscillatory state (from section 3.3) as the control state. We observe that a critical factor in simulating the condition of Schizophrenia is the overall decrease of the membrane conductance in the network. All AMPA neurotransmitter receptor related synaptic conductances in L4 and L6 (but not LGN) are reduced by 7% from their base values; all GABA_*A*_ neurotransmitter receptor related conductances are reduced by 10% from their base values. Next, the synaptic connectivity changes are made as shown in Figure 5A. Compared to Figure 4C(i), there is a decrease in the feedforward connectivity from the TCR to the Py4 and SS4 populations by ≈ 5 and 3%, respectively.

First, we tested the model with noise input to the LGN. In Figures 5B(i),(ii), we can see a significant increase in the power within the θ band (*P* < 0.001) and a simultaneous siginificant decrease of the β band power (*P* < 0.001). This is in agreement with similar observations made in the experimental study on Schizophrenia patients by Adams et al. (6), although both controls and patients were in the awake resting state with α peak oscillations. In the model presented here, if the control condition is set to represent the awake resting state with α peak oscillations, we observe a decrease in power within the β band, but not the simultaneous increase in power within the θ band. We have not presented these observations here, as we plan to continue along these lines in ongoing work (see section 4 for a discussion).

Next, we wanted to validate the β band changes reported in the SSVEP of Schizophrenia patients by Giri et al. (16), where they have shown reduced SSVEPs for 17, 23, and 30 Hz of flicker stimulation relative to controls. Here, we have presented our observations corresponding to a periodic input of 17 Hz with additive noise to the LGN. This simulates condition of SSVEP in our *in silico* model. In Figure 5C(i), we observe a significant decrease (*P* < 0.001) in β band power similar to that reported by Giri et al. (16). The line plots in Figure 5C(ii) shows the SSVEP first harmonic peak at the input frequency of 17 Hz, demonstrating entrainment and the condition of SSVEP simulated in this *in silico* model as reported in our previous work (12). The power of the first harmonic is lesser for Schizophrenia patients. In addition, we also see a significant increase (*P* < 0.001) in the theta band power as reported in other studies (see above), but not by Giri et al. (16). We have also tested for other β input frequency values (20 & 23 Hz), not shown here for brevity. Our results are consistent across all tested input frequencies.

## 4. Discussion

The importance of understanding the synaptic connectivity correlates of brain oscillatory changes cannot be more emphasized than in neurological disorders (25, 26). As brain rhythms are impacted during neurological conditions, they become an important neuromarker for the first line of clinical investigation (27). For example, slowing of the frequency spectra recorded in EEG is a definitive neuromarker of Alzheimer's disease, a condition inflicted by aggressive loss in brain synaptic connectivity (2, 7); dysfunctional beta oscillations due to anomalies in the networks of GABA interneurons and in cortico-cortical connections are associated with cognitive deficits in Schizophrenia (9, 28, 29). Therefore, it becomes critical to understand the exact synaptic pathway correlates of such neuromarkers, and possible therapeutic solutions. One clinically successful instance of therapeutic solution for the EEG neuromarker (unwanted 10 Hz oscillation) of Parkinson's disease is Deep Brain Stimulation (DBS) (30). However, clinical investigation of possible therapeutic treatments is hard to conduct in neurological patients; this is especially problematic in instances of cognitive impairment, where patients may feel distress, which in turn may aggravate their condition. Animal models of neurological conditions have provided a lot of clues, but human and primate brains react differently than animals, and findings may be misleading. There are other organoid solutions being suggested recently (31). One approach that is relatively cheaper and have low demands on resources are *in silico* models that are brain inspired. Thus, these models can be used to represent neural circuitry governing brain rhythms and deepen our understanding of healthy as well as sick brain states. They also come as an important aid in exploring new, as well as enhancing existing, therapeutic treatments for neurological conditions (10, 11).

We have been using *in silico* models of the thalamocortical loop, informed by anatomical and physiological studies of the brain visual pathway, to simulate brain rhythms within the alpha and theta band frequencies. Such frequency domain analysis of brain signals such as EEG, SSVEP and LFP are by far the most popular means to visualize and correlate specific frequency sub-bands [*viz*. delta (δ), theta (θ), alpha (α), beta (β), and gamma (γ)] to brain states in both health and neurological conditions. In a recent work, we have validated a model with psychophysical studies that demonstrated phase entrainment of brain signal during SSVEP. In this work, we have presented an enhanced version of this model by adding more biologically plausible synaptic layout and connectivity parameters. Our *in silico* model consists of three modules *viz*. the lateral geniculate nucleus (LGN), that is the visual thalamus receiving direct input from the retina; Layers 4 and 6 of the primary visual cortex (V1), both of which receive feedforward input from the LGN; the L6 provide feedback to the LGN. The model synaptic layout is as in Sherman (15). The synaptic parameters for intra- and inter-layer connectivity in L4 and L6 are computed from Binzegger et al. (14). The intra-layer connectivity in LGN and feedback from V1 layers are as in our previous works (2). For missing data in our sources, informed assumptions are made. The voltage time series outputs of the model neuronal populations are converted to the frequency domain to identify the dominant frequency of oscillation. The outputs of the excitatory projection neurons of each module, *viz*. the Thalamocortical Relay (TCR) cells of the LGN, and Pyramid cells of L4 (Py4) and L6 (Py6) are observed for demonstrating brain state transitions. Where we have validated our results with clinical data, the output of the L4 is considered. This is in agreement with the observations made in Maier et al. (19), where the current source density (CSD) of LFP recordings from V1 following a visual stimulus had origins in L4 and was attributed to the thalamocortical afferents.

One approach for investigating clinical conditions with *in silico* model is to set a “control” state that will simulate the specific brain state (which the model is intended to investigate) in healthy adults. This control state acts as a reference point from where model parameters and attributes that produce aberrations corresponding to the disease states can be identified. We have adopted a similar approach in this work. At first, we recorded the base state oscillations of the model that emerged when the synaptic connectivity parameters in the model were set to the base values mentioned in Table 3. Next, starting from the base state of the model, we simulated transitioning into the α, β, and θ states, and identified the underpinning synaptic parameter pathways. Note that for each state, we aimed at all the excitatory projecting populations, *viz*. the TCR of LGN, Py4 of L4 and Py6 of L6 to have a peak power within the respective bands. This is because, to the best of our knowledge, there is no coherent data in literature that suggest the possible oscillatory combinatorics in LGN and V1 and their associated brain states. Furthermore, literature suggest that same areas of brain may employ multiple mechanisms to generate multiple frequency bands based on the brain state and function (32). We too have observed an exploding parameter and oscillatory state combinatorics in the model. We have chosen to demonstrate the synaptic connectivities that can produce the same peak oscillatory frequency in the TCR, Py4 and Py6 populations simultaneously.

In the base state of the model, the peak frequency of oscillation for TCR and Py6 is in the α band, and for Py4 is in the θ band. For long, the thalamus has been attributed as the primary pacemaker of α oscillations (33, 34). However, LFP recordings from the V1 of macaque monkeys and dogs suggest α oscillation origins in the deep cortical layers like Layer 5 (L5) (35, 36) and L6 (37). Another LFP based study of the occipital lobe of macaque monkeys suggest the superficial cortical layers 2/3 (38) as the origin of α rhythms. Bollimunta et al. (37) and Mila et al. (38) also discuss the possibility of separable thalamic and cortical α pacemakers which become differentially active and coupled under different behavioral conditions. Interestingly, two mesoscopic computational models where the α oscillations can be generated with or without the thalamic drive is reviewed in Sigala et al. (39). To understand the origin of the α rhythm in our base state network, we lesioned the thalamus from the V1 layers by disconnecting all synaptic feedforward and feedback pathways from and to the LGN, respectively. All other connections were left intact. This lesioning did not affect the α oscillations in Py6; rather, the TCR α peak moved from the α high (α-H) to the α low (α-L) sub-band due to lesioning the Py6 feedback. Thus, our model supports the discussions in Bollimunta et al. (37), Mila et al. (38) where both LGN and the infragranular L6 in V1 demonstrate α rhythm generation independently. In future works, we have plans to integrate the infragranular L5 and supragranular L2/3 in our model, which will allow us to test this aspect more thoroughly.

In addition to the above, lesioning of the LGN also did not affect the Py4 peak frequency within the θ band. To understand the origins of this θ oscillations, we lesioned the L4 and L6. The Py4 peak frequency shifts to the α band, but the Py6 peak frequency moves from α-L to α-H. Investigating further, we identified that the inhibitory feedback from Py6 to Py4 is the primary cause of the θ dominance in Py4. In turn, the feedforward excitation of L4 on L6 slows down the α band power in Py6.

Next, from the intact base network state, we aimed at identifying synaptic connectivity parameters that could cause brain state transitions to θ, α, and β bands for TCR, Py4 and Py6 simultaneously. Our findings are as follows:

- Theta: Increased excitation from L4 to L6 caused a shift of peak frequency from α to θ in Py6, which in turn caused the TCR output to move from the α band into the θ band. Selective lesioning in this state confirmed that the L4 drives this θ rhythm state of the network. We did not find any anatomical study to support this finding, and leave this as a testable attribute identified by the model.
- Alpha: The TCR and Py6 were already oscillating within the α band. Decreasing the inhibitory projections from L6 to L4 shifted the L4 dominant frequency to the α band. We have already discussed (see above) the agreement of our observations with anatomical studies.
- Beta: To generate β rhythm in our model, the following changes to synaptic connectivity were made in the base state: the TCR to L4 excitatory projections as well as the self-excitatory loop in L4 were increased; the inhibitory feedback from the L6 to L4 were decreased; (these three changes led to an overall increase in the excitation in L4); the Py4 to Py6 excitatory projection was increased; (as L4 was already in an increased excitatory state, this led to an overall increase in excitation of the Py6 too); last, the feedback from Py6 to the inhibitory populations of LGN was decreased; (this resulted in disinhibiting the TCR, which in turn led to increased excitatory efferents to L4, and onwards to L6). While the overall increase in the excitatory state of L4 is essential to shift the Py6 oscillations to the β band, the Py6 feedback is critical for the β band transition in LGN; if these feedback connections are lesioned, the TCR oscillations shift out of the β band. On the other hand, if the LGN feedforward path to the L4 is lesioned, the β band oscillations in the L4 vanishes. Thus, our study indicates that the β band originates at infragranular layer L6, which then propagates to the LGN and L4.
  A study of rat somatosensory and auditory cortex showed 20–30 Hz (β high (β-H) band) oscillations to be characteristics of the infragranular layers (40, 41). Also whole-cell *in vivo* recordings of rat visual cortex found the β low (β-L) band frequencies to be most pronounced in L5 (42). A recent review on cortical layers and rhythms, carried out in René and Pascal (43) supports the findings of β rhythm in infragranular layers. Thus, our above-mentioned observations agree with the theory of cortical β rhythm originating in the infragranular layers. At the same time the overall thalamocortical loop helped maintain the β rhythm in the Py6, which agrees with observations mentioning the thalamocortical circuitry playing a role in maintaining the cortical rhythms across its laminar structures (5).

This concludes our study of the control state conditions of the model in the θ, α, and β band oscillatory state.

As a case study, we demonstrate the known alterations in θ and β band frequencies in the condition of Schizophrenia. It is reported by Adams et al. (6) that resting state EEG of Schizophrenia patients show a decrease in β band power and increase in θ band power compared to controls. (They have also mentioned alterations in the γ band, but we do not consider the γ band oscillations in this work). At the same time, the authors make a computational study to validate their psychophysical results and propose possible synaptic correlates of the frequency band alterations in Schizophrenia conditions. Overall, they identified several combinations of excitatory-inhibitory imbalances that simulated the β and θ band power alterations in their model. Similar combination of psychophysical and animal model-based studies also indicate the excitation-inhibition imbalance as the neuronal correlates of the β band alterations observed in Schizophrenia patients compared to controls (9). We observed that two critical changes in our model induced a significant decrease and increase (with *P* < 0.001) in β and θ band power, respectively: (a) decrease in the AMPA and GABA_*A*_ synaptic conductance parameter values; (b) decrease in the excitatory synaptic connectivities from LGN to L4. The observations in (a) conform to those in the above-mentioned researches (6, 9). The observations in (b) are particularly interesting, where visual pathway impairment is identified by many researches as a biomarker of Schizophrenia (44). Along these lines, an SSVEP study by Giri et al. (45) demonstrated significant decrease (*P* < 0.001) within the β band power in SSVEP outputs corresponding to selected visual flicker stimuli. Tested with our model for simulated conditions of Schizophrenia, the results agree with these observations.

Readers may note that the condition of Schizophrenia in our work is simulated with the β band oscillatory model state as the control condition. However, in Adams et al. (6), the EEG studies were made when all controls and patients were in the awake resting state and showing an α peak oscillation. While we could generate a reduced β band power when the model is in the α oscillatory control state, we did not see an increase in the θ band power, thus unable to fully validate the experimental results in the model. The main criticism of the model presented here is that the synaptic as well as the structural layout of the V1 module is simplified. Also, synaptic connectivity parameters in the model are informed by the cat visual cortex, but the Schizophrenia symptoms that are being validated are observed in humans; this is in spite of the primate visual system having evolved with significant differences compared to cats. As a future work, we will enhance the V1 model by adding the superficial L2/3 and infragranular L5 and parameterizing based on primate anatomical and physiological data (46). Furthermore, we aim to include γ rhythm observations in the model, as these are also identified as possible neuromarkers in the EEG of Schizophrenia condition. Also, we plan to use cross-frequency coupling (CFC) measures to analyse our model dynamics, as CFC allows a better understanding of thalamus and neocortex information processing (47) and abnormal behavior in neurological diseases (48).

The *in silico* model presented here belongs to the neural mass genre of computational models (NMM) that can simulate complex biological phenomena with fewer state variables and parameters. This make NMMs computationally less expensive compared to spiking neural networks (SNN), although at the cost of single-neuron-level dynamics unlike in the latter (49). In addition, the sigmoid function used in NMMs to represent the neuronal activity for a population is not based on a detailed biophysical description of spiking neuron (50). Subsequently Byrne et al. (50) suggested a change in NMM tactics and proposed a next generation NMM. Recently, Huang and Lin (51) proposed a current density based model of NMM. Interestingly, Ruffini et al. (52) proposed a laminar based NMM for transcranial current stimulation (tCS). tCS is emerging as a promising alternative treatment in drug resistant Schizophrenia patients (53) and hence modeling this use case gives opportunity to personalize the treatment for individual patients. As future work, we see potential to extend the Schizophrenia case-study presented here to better understand the effects of tCS in the condition.

## 5. Conclusion

In conclusion, we have tested a simple *in silico* neuron population network of the thalamocortical loop in the brain visual pathway. The model could generate brain rhythms within the α, θ and β bands and correlate with specific synaptic connectivities and pathways. As a case study, we have simulated the oscillatory power alterations as observed in experimental studies on Schizophrenia patients. Our results show a significant (*P* < 0.001) decrease in beta band power and a simultaneous increase in the theta band power, similar to that observed in Schizophrenia.

The main contribution of this work is a thorough analysis of the synaptic connectivity parameter combinatorics that generates the α, θ, and β oscillatory states in the *in silico* model; for brevity, we have presented only selected combinations in this work. Findings from anatomical and physiological studies suggest that all three oscillatory states are observed in both LGN and the V1. It is however hard to identify the exact synaptic connectivity combinations that underpin the continuum of changing brain states *in vivo*. Our findings demonstrate the potential for designing and using simple brain inspired neural networks to simulate both control and neurological conditions. This in turn has the potential to be used for investigating individual therapeutic requirements to reverse or suppress those conditions. As ongoing work, we are developing this model to allow deeper insight into the synaptic connectivities and attributes that underpin the conditions of Schizophrenia.

## Data Availability Statement

The original contributions presented in the study are included in the article. Further inquiries can be directed to the corresponding author/s.

## Author Contributions

SS: methodology, software, investigation, writing—original draft, and writing—review and editing. BS: conceptualization, methodology, writing—original draft, writing—review and editing, and funding acquisition and supervision. Both authors contributed to the article and approved the submitted version.

## Funding

This research was supported by the Birla Institute of Technology and Science (BITS) Pilani Goa Campus Grants BPGC/RIG/2018-19 and GOA/ACG/2019-20/Oct/02 to BS. SS was supported by the BITS Pilani Goa Campus Institute Fellowship awarded toward her Doctoral Research.

## Conflict of Interest

The authors declare that the research was conducted in the absence of any commercial or financial relationships that could be construed as a potential conflict of interest.

## Publisher's Note

All claims expressed in this article are solely those of the authors and do not necessarily represent those of their affiliated organizations, or those of the publisher, the editors and the reviewers. Any product that may be evaluated in this article, or claim that may be made by its manufacturer, is not guaranteed or endorsed by the publisher.
